# How a simple medical observation led to increased awareness and propagation of research in Kawasaki disease in Egypt

**DOI:** 10.21542/gcsp.2017.18

**Published:** 2017-10-31

**Authors:** Galal El-Said, Sherif Rizk, Khaled Sorour, Soliman Gharib, Karim Said, Hossam Kandeel

**Affiliations:** Cairo University, Cairo, Egypt

## Introduction

Kawasaki disease (KD) is a serious vasculitis of children first described by Tomisaku Kawasaki five decades ago.^1^ Its etiology is unknown, which led to the miasma (bad air) theory of disease as a possible explanation; this theory of disease was accepted in ancient times pointing to the existence of microorganisms unseen by human eyes as the cause for many disease of unknown etiology.^2^ So investigators started to look for airborne pathogens which could be the culprit for causing KD.^3^

It is an inflammatory self-limited condition lasting for an average of 12 days without treatment and occurs in children usually before the age of 5 years. Boys are affected more commonly than girls. If left untreated in its acute phase it may result in significant complications and mortality, causing several cardiovascular complications including coronary artery aneurysms, cardiomyopathy, heart failure, myocardial infarctions, arrhythmias, sudden death and peripheral arterial occlusions.

KD is most prevalent in Asian children or Asian immigrants living in other countries.^4^ Most large epidemiological studies came out of East Asia, followed by the United States and Western Europe, where KD is the most common cause of acquired heart disease after the near eradication of rheumatic fever and rheumatic heart disease.^5^ Several authors have noticed an apparent increased frequency of KD cases worldwide. Possible reasons, in addition to a true increase in frequency, include improved diagnosis, better reporting and inclusion of incomplete cases in the total.^6^ Although this disease is serious with important complications of the cardiovascular system if not diagnosed early; the incidence of KD is unknown in Egypt as rheumatic fever and rheumatic heart disease are still the commonest cause of acquired heart disease in children. The first time KD was reported from the Middle East and Africa was of an Egyptian child living in Saudi Arabia.^7^ Soon other reports followed from different countries of the region.

## Coronary artery ectasia

It was noticed that coronary ectasia (CE) (by definition an abnormal enlargement of a coronary artery ≥1.5 times the adjacent normal segment) is common among Egyptian patients with CAD during their coronary angiograms.

A retrospective study from the Texas Heart Institute^8^ was conducted on 275 Egyptian patients treated for coronary artery disease (CAD) between 1980-1995. Clinical, demographic and angiographic data were reviewed. Forty-five patients (16.4%) had CE and were compared to the remaining 230 patients without. This is a higher incidence of CE than is reported from other patient populations, 0.3 to 5%.^9,10^

Coronary risk factors and angiographic data were compared between the groups. Among the important angiographic data collected was the anatomic distribution and extent of ectasia in the coronary arterial tree. In this study, patients with CE were more commonly obese (*p* < 0.01) and had a higher incidence of multiple-vessel coronary artery disease, including left main disease (not statistically significant). Although hypertension^11^ and dyslipidemia^12^ have been associated with CE in other studies, in this study there was no difference in the classic CAD risk factors between the CE and non-CE groups. Atherosclerosis, KD and aging were discussed as possible causes of ectasia, but the authors concluded that the high incidence of CE in Egyptian patients with CAD maybe due to a more aggressive remodeling process of the atherosclerotic vessel and that obesity may play a role. Some of these patients may have been due to antecedent KD but although the authors mentioned KD as possible cause, they were not well oriented about this disease at that time.

## Kawasaki disease in Egyptian adults

In 2011, one of the authors of this paper (Prof. Galal El-Said) among others, felt that many of these cases could be missed KD, but there was no study to corroborate this observation. It was not until Drs. Jane Burns and John Gordon gave important talks in Egypt, that physicians were alerted that coronary artery aneurysms (CAA) that lead to ischemia and infarction in adolescents and adults may be due to antecedent KD. This aroused the interest in the diagnosis and management of this serious disease, especially with the decrease in the incidence of rheumatic fever and measles in Egypt. Cases of KD were diagnosed more often in pediatric hospitals and articles started appearing in international literature as case reports.^7,13^

The aroused interest helped us to plan for a study similar to one conducted in San Diego with her colleagues, which showed that KD in Egypt is not uncommon, but missed. Angiographic findings that make antecedent KD likely include proximal aneurysms with or without calcification, associated with angiographically-normal distal segments.^14^ Because a history of KD may be difficult to obtain from young adults who might have been too young to have a personal memory of the illness, recent guidelines from Japan recommend that patients with acute coronary syndromes and aneurysms be diagnosed as having sequelae of KD if other conditions causing aneurysms such as atherosclerosis and collagen vascular disease are excluded.^15^

**Figure 1.**
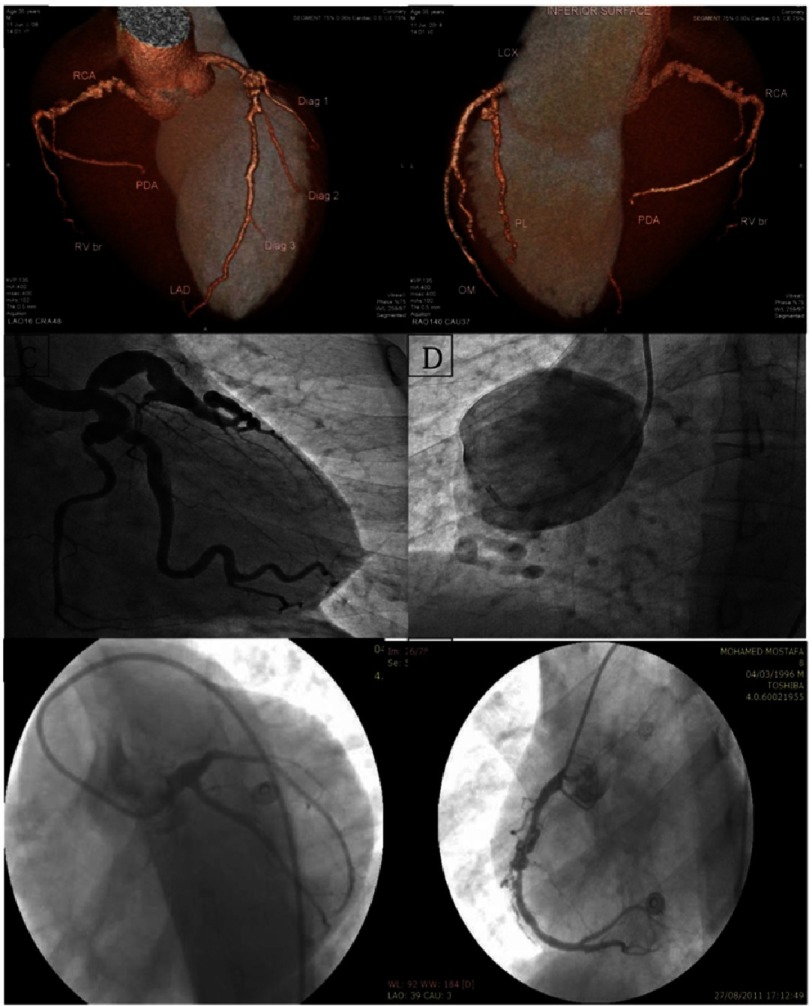
Examples of angiograms of definite and probable KD cases. A & B: MSCT (reconstructed image) of a 35-year-old male patient who presented with unstable angina, showing proximal LAD (9 mm), mid LCX (7 mm), and proximal RCA (7 mm) aneurysms with angiographically normal distal segments. C & D: Coronary angiogram of a 25-year-old male patient presenting with typical chest pain at rest, showing proximal LAD, LCX, and obtuse marginal branch (OMB) aneurysms (left image) and proximal RCA 54 mm aneurysm (right image). E & F: Coronary angiogram of a 14-year-old male patient with history of missed KD in childhood presenting with anterior myocardial infarction, showing proximal LAD total occlusion by a thrombus (left image) and proximal and mid-RCA aneurysms with thrombus in the more distal aneurysm (right image).

In this study conducted in Egypt by Rizk et al.^16^, a total of 580 angiograms (conventional and multi-slice CT), of patients aged 40 years or younger from the three centers were reviewed by Dr. John Gordon and Dr. Lori Daniels from San Diego. Coronary artery aneurysms were reported in 46 cases (7.9%). The majority of patients were male. The most commonly encountered risk factors were smoking, followed by dyslipidemia. The group adjudicated as equivocal had more traditional cardiovascular risk factors compared to the definite and probable groups combined. Indications for angiographic evaluation of these 46 patients with aneurysms were as follows: nine had myocardial infarction with elevated troponin-I levels (including five with inferior ST-segment elevation myocardial infarction (STEMI), two with anterior STEMI, and two with non-STEMI), while the remaining patients had angina (23 with unstable angina and 14 with exertional angina either not responding to medical treatment or confirmed by positive stress test).

Of the 46 patients with aneurysms, ten (22%) were adjudicated as definitely due to antecedent KD. All had a history of KD or a KD-compatible illness (history of classic KD that was misdiagnosed in three, scarlet fever in one, and measles in six). One of the three patients with missed KD was a 14-year-old male presenting with an anterior ST-segment elevation myocardial infarction (Figure 1). Seven years previously he had presented with clinical criteria for KD but was misdiagnosed with acute rheumatic fever. Echocardiography at presentation showed no coronary artery abnormalities but a repeat echocardiogram three months later revealed aneurysms of the proximal right and left coronary arteries.

An additional 29 patients had no history of a KD-compatible illness but had aneurysms adjudicated as probably due to antecedent KD based on their proximal location, and angiographically-normal distal vessels without changes suggesting atherosclerosis. Of these 29 patients, 24 patients underwent MSCT and 8/24 (33%) had calcification of their aneurysms. Seven of the 46 patients (15%) were adjudicated as equivocal because their aneurysms were diffuse, with or without significant luminal narrowing suggestive of atherosclerosis. The median size of the largest aneurysm was 9.0 mm (7.0–12.0) for the definite group, 7.5 mm (6.5–54.0) for the probable group, and 6.5 mm (6.0–7.5) for the equivocal group (*p* = 0.03 for the difference between definite and probable groups vs. equivocal group). Assessment of the distribution of coronary arteries affected by aneurysms revealed that the left anterior descending artery was the most commonly affected artery followed by the right coronary artery.

The distribution of patients with one, two, or three coronary arteries with aneurysms differed by the group classification. Of the ten patients with only one coronary artery affected, five (71%) were classified as equivocal, four (14%) were probable, and only one (10%) was definite. Of the 36 patients with two or more coronary arteries affected, only two (29%) were classified as equivocal, 25 (86%) were probable, and nine (90%) were definite (*p* = 0.003). Giant coronary artery aneurysms (≥ 8 mm) were present in 22 patients (48%), and eight patients (36%) had thrombi in their coronary aneurysms.

Acute management of the 46 patients was as follows: all patients received aspirin, a β-blocking agent, nitrates, and statins. Patients with hypertension or myocardial infarction also received an angiotensin converting enzyme inhibitor. Six patients underwent percutaneous transluminal coronary angioplasty with stent placement, four had angioplasty alone, and 21 patients were started on oral anticoagulation with warfarin. One patient underwent surgical excision of a 54 mm right coronary artery aneurysm with interposition of a saphenous vein graft (Figure 1). Histologic examination of the excised right coronary artery aneurysm revealed moderate degenerative changes with mild hyalinosis and loss of the elastic lamina.

In this study, 6.7% of young adults who underwent angiography to evaluate symptoms of suspected myocardial ischemia had coronary artery aneurysms that may have been due to antecedent KD, highlighting the seriousness and importance of early KD diagnosis and management to avoid its serious cardiovascular sequalae - most importantly, coronary artery aneurysms. In Egypt it seems KD is more common than was anticipated before and was frequently missed. This raises the possibility that KD is not uncommon in Egypt where other pediatric rash/fever illness such as measles, scarlet fever, and acute rheumatic fever are still prevalent.

Based on this study, clinical characteristics that make antecedent KD more likely include fewer traditional cardiovascular risk factors, aneurysms in more than one coronary artery, and the presence of proximal giant coronary artery aneurysms. The majority of cases in this series were men and KD is known to have a male predominance with more severe outcomes in male children.^17,18^

Smoking has been noted as a prominent risk factor among young adults with a history of KD presenting with myocardial infarction, which suggests a possible acceleration of ischemic complications in this subset of KD patients.^18^ This was the first study in the Middle East to systematically evaluate a population of young adults undergoing coronary angiography to estimate the prevalence of missed KD as a potential contributing factor. In Japan, the country with the highest incidence of KD, Kato et al. surveyed adult cardiologists and retrospectively identified 130 patients, aged 20 to 63, with angiographic and clinical findings suggestive of KD.^19^ Although a definite history of KD was elicited in only two patients, the authors concluded that all 130 patients were likely to have had KD as the cause of their cardiovascular abnormalities.

The findings in this study were also consistent with findings described by Daniels et al. in San Diego, who evaluated a similar population of young adults from the US. By reviewing the medical history and coronary angiograms of all adults under age 40 who underwent coronary angiography for evaluation of suspected myocardial ischemia at four San Diego hospitals from 2005-2009, they found that 5% had aneurysms definitely (*n* = 4) or presumed (*n* = 9) secondary to KD as the etiology of their coronary disease. Because of the unique therapeutic challenges associated with these lesions, adult cardiologists should be aware that coronary artery aneurysms in young adults may be due to missed KD in childhood.^20^

Another study using IVUS and FFR to differentiate between atherosclerotic and KD coronary artery aneurysms is currently in progress in Egypt. This has led to increased awareness of KD among Egyptian pediatricians and physicians with emerging reports of atypical KD patients with the latest report of a 12-year-old male adolescent presenting with meningitis as a complication of incomplete KD, and the authors concluded that a high level of suspicion is required for early diagnosis and management of Kawasaki disease irrespective of the clinical presentation or ethnicity to avoid missing the diagnosis and decrease the incidence of CAAs.^21^

The interest in KD is increasing which led Sir Magdi Yacoub to dedicate a whole issue of this Journal to KD for better awareness of the seriousness of this disease.
